# Mechanisms of miR-18a-5p Target NEDD9-Mediated Suppression of H5N1 Influenza Virus in Mammalian and Avian Hosts

**DOI:** 10.3390/vetsci12030240

**Published:** 2025-03-03

**Authors:** Jipu Wang, Yanan Xing, Lin Chen, Shuyi Han, Ye Wang, Zhilei Zhao, Gaojian Li, Wenchao Li, Hongxuan He

**Affiliations:** 1 Anhui Province Key Laboratory of Animal Nutritional Regulation and Health, College of Animal Science, Anhui Science and Technology University, Fengyang 233100, China; keke0102@yeah.net; 2 CAS Key Laboratory of Animal Ecology and Conservation Biology, Institute of Zoology, Chinese Academy of Sciences, Beijing 100101, China; xingyanan@ioz.ac.cn (Y.X.); hanshuyi13@163.com (S.H.); yew4315@126.com (Y.W.); zhaozhilei@ioz.ac.cn (Z.Z.); ligaojian22@ioz.ac.cn (G.L.); 3 University of Chinese Academy of Sciences, Beijing 100101, China; 4 National Center for Nanoscience and Technology, Beijing 100101, China; chenl@nanoctr.cn

**Keywords:** miR-18a-5p, H5N1, NEDD9, mammal, poultry

## Abstract

MicroRNAs (miRNAs) are key regulators of gene expression, exerting post-translational control through mRNA silencing or degradation. These molecules play pivotal roles in host–pathogen interactions, particularly in modulating antiviral immune responses. The global public health threat posed by the H5N1 Highly Pathogenic Avian Influenza (HPAI) virus necessitates urgent exploration of novel therapeutic strategies. Our investigation revealed significant dysregulation of miR-18a-5p following influenza virus infection, observed consistently across both in vitro and in vivo models. Experimental evidence demonstrated that miR-18a-5p overexpression effectively inhibits H5N1 virus propagation through multiple mechanisms: (1) in vitro studies using A549 cells transfected with miR-18a-5p mimics showed a substantial reduction in viral replication; (2) animal models (mice and chickens) with elevated miR-18a-5p expression exhibited markedly suppressed AIV replication, reduced pathogenicity, and improved survival rates. The therapeutic potential of miR-18a-5p was particularly evident in its ability to significantly decrease mortality rates in H5N1-infected animals. Furthermore, this miRNA demonstrated robust protective effects against virus-induced lung damage, suggesting its dual role in both preventing and treating H5N1 infections. These findings position miR-18a-5p as a promising candidate for the development of broad-spectrum antiviral interventions, offering a novel strategic approach to combat this serious public health challenge.

## Simple Summary

This study found that a small molecule of RNA called miR-18a-5p behaved abnormally when infected with the H5N1 avian influenza virus. By increasing the amount of this RNA in the laboratory, the researchers found that it can effectively inhibit the replication of the H5N1 virus in cells and in mice and chickens, reducing the pathogenicity and mortality of the virus. This suggests that miR-18a-5p may be a new method for the prevention or treatment of H5N1 virus infection.

## 1. Introduction

H5N1 has the ability to infect mammals across species, and its transmission is mostly due to close contact with or consumption of infected birds. Continued mutations of the virus may lead to an increased risk of human-to-human transmission. The effective prevention and control of infections present significant challenges due to the potential for continuous transmission by asymptomatic carriers and the risk of vaccine resistance arising from breakthrough transmissions. Evaluating the inhibitory effect of H5N1 viral replication will provide an important basis for the development of novel antiviral therapies. The objective of our study was to elucidate the mechanism via which miR-18a-5p suppresses viral replication in mammals and birds, enabling the development of more effective antiviral therapies.

The avian influenza virus (AIV), classified within the Orthomyxoviridae family, possesses a segmented genome consisting of eight single-stranded RNA fragments with negative polarity [1]. The classification is determined by the antigenicity of the surface glycoproteins, namely, hemagglutinin (HA, with subtypes H1–H18) and neuraminidase (NA, with subtypes N1–N11). Type A influenza viruses (AIVs) are classified into different subtypes (e.g., H5N1, H7N9, H9N2, etc.) [2]. Except for H17N10 and H18N11, most of the subtypes of the influenza virus can be isolated from wild aquatic birds [3]. Infections of AIVs are generally species-specific. AIVs mainly infect birds with significant morbidity and mortality. Occasionally, AIVs can spillover into humans and other mammals, in which they can cause a range of consequences, from mild infection to lethal infection [4,5]. Over the past few decades, AIVs (subtypes such as H5N1, H5N2, H7N9, H9N2, and H10N8) have caused multiple cases of human infection and have a fairly high mortality rate. Especially between January 2022 and September 2023, there have been at least 12 reported human infections with clade 2.3.4.4b A(H5N1) viruses [6]. There is currently no conclusive evidence that naturally occurring avian influenza viruses (AIVs) have acquired the ability to transmit effectively or sustainably from person to person in the population. The reason for concern about HPAIVs is their potential capacity to cause a pandemic in human populations. Due to genetic combination and genetic reassortment, novel subtypes of AIV frequently arise, causing repeated outbreaks around the world [7]. The continuous evolution of AIVs promotes their potential to cause a pandemic [4].

AIVs mainly infect epithelial cells of the upper and lower respiratory system, while host cells have developed microbial pathogen recognition systems (PPRs) to recognize a broad spectrum of pathogen-associated molecular patterns (PAMPs) [8]. During the course of infection, AIVs encounter numerous bottlenecks constituted by host antiviral innate responses initiated by PPRs, including production of cytokines, activation of autophagy, regulation of signaling cascades, modulation of miRNAs, etc. [9,10,11]. AIVs encode about 17 proteins with an eight-segmented RNA genome. This limited coding capacity demands that AIVs manipulate cell functions extensively by any achievable mechanisms to combat the restriction factors constituted by host cells [12], such as utilizing cellular transport systems, exploiting protein synthesis systems, manipulating signaling transduction networks, etc. [13,14].

Although the study does not clearly articulate the specific mechanism of action between NEDD9 and severe acute respiratory syndrome coronavirus 2 (SARS CoV-2), which causes COVID-19, it provides a direction for further research into the relationship between NEDD9 and the virus. NEDD9 plays an important role in a variety of diseases, and although there are no direct studies elucidating its role in viruses [15,16], the following analysis and speculations can be made from the perspective of related diseases and mechanisms: Chronic hepatitis B virus (HBV) infection is one of the main causes of primary liver cancer (HCC) [17]. The expression level of NEDD9 in HCC cells is significantly increased. Its high expression promotes the migration and invasion of HCC cells and is closely related to tumor growth, infiltration, and metastasis. Although there is no direct evidence to show that NEDD9 has a direct interaction with HBV, in the process of liver cell lesion formation and tumorigenesis caused by HBV infection, NEDD9 may promote the transformation of liver cells infected by HBV into tumor cells and the development process of tumors by participating in cell signaling pathways [18,19]. NEDD9 is a key regulatory factor for lymphocyte migration. During viral infection, the migration of immune cells is crucial for the body to recognize and eliminate viruses. For example, in HIV infection, NEDD9 may affect the migration and homing of immune cells such as CD4+ T lymphocytes, thereby indirectly affecting the body’s immune response to the HIV virus. If the function of NEDD9 is abnormal, this may lead to the inability of immune cells to effectively migrate to the virus-infected site, affecting immune surveillance and clearance functions [20]. In summary, although there are no direct studies elucidating the role of NEDD9 in viruses, it can be seen from the potential association of NEDD9 in a variety of diseases and COVID-19 that NEDD9 may play a potential role in the development of diseases caused by viral infection. More research is needed in the future to delve into the specific relationship between NEDD9 and the virus.

As a class of non-coding single-stranded small RNAs, microRNAs (miRNAs) play an important role in gene regulation, animal immune response, and antiviral processes in animals [21]. MiRNA is an endogenous, small single-stranded non-coding RNA molecule, generally 21~23 nt in length, with a highly conserved region, which mediates gene regulation through the target site of the 3′-untranslated region (3′-UTR) of the target mRNA through the guide protein argonaute (AGO) [22]. MiRNAs are biomarkers that have attracted much attention in recent years; for example, microRNA-376b-3p promotes the replication of porcine reproductive and respiratory syndrome virus by targeting the viral restriction growth factor TRIM22 [23]. MiR-18a may affect the replication and transcription processes of hepatitis B virus (HBV) by targeting the genome of HBV or key factors related to HBV replication and transcription in host cells, such as certain transcription factors and signaling pathway proteins [24,25]. The role of miR-18a in viruses is diverse and complex. Beyond its established functions in hepatitis B virus regulation, this molecule also demonstrates significant involvement in other viral pathways. Notably, the microRNAs miR-18a and miR-452 have been shown to modulate enterovirus 71 replication through specific targeting of the VP3-encoding gene [26]. Through compromising the DNA damage response mechanism, miR-18a facilitates Epstein–Barr virus reactivation and contributes to chromosomal instability in virus-associated lymphomagenesis [27].

However, there are currently no clear reports on the relationship between miR-18a and H5N1, and the idea of the experiment performed in this study was to explore whether miR-18a-5p affects H5N1 replication or host antiviral response by targeting specific genes or pathways, with the aim of identifying new therapeutic targets and developing innovative antiviral strategies. This study will contribute to a deeper understanding of host–virus interactions and provide insights for the prevention and control of AIV outbreaks.

## 2. Materials and Methods

### 2.1. Ethics Statement and Biosafety

The experimental procedures involving animals were strictly conducted in compliance with the US National Institutes of Health guidelines for laboratory animal care and use, following ethical approval from the Institute of Zoology, Chinese Academy of Sciences (IOZ-IACUC-2022-250). All influenza virus-related experiments were performed within the Animal Biosafety Level 3 (ABSL-3) containment facility at the Research Center for Wildlife Diseases, which is officially authorized by the Chinese Academy of Sciences.

### 2.2. Cell Lines, Viruses, and Animals

A549 cells (human airway epithelial cell line) and 293T cells (human renal epithelial cell line) were obtained from the ATCC and maintained in DMEM (Dulbecco’s modified Eagle medium; Gibco, Thermo Fisher Scientific, Cambridge, UK) supplemented with 10% fetal bovine serum under standard culture conditions of 37 °C and 5% CO_2_. Influenza A QH1/H5N1 (A/environment/Qinghai/1/2008(H5N1)) viruses were propagated in 10-day-old specific pathogen-free (SPF) chicken embryos (Vital River Laboratories, Beijing, China). Virus titers determined for infection were calculated by plaque assay or TCID50 titration on MDCK cells.

BALB/c mice were purchased form HFK Bioscience (Beijing, China) and maintained in the Institute of Zoology, Chinese Academy of Sciences.

### 2.3. Extraction of RNA

Infection of cells and extraction of RNA method: dilute QH1/H5N1 influenza virus and gently add 100 μL of virus dilution to each well to infect A549 cells at MOI = 1. Gently shake the cell culture dish so that it evenly coats the cells. Each dilution requires 3 parallel wells to be set. Then, add 0.5 mL of viral culture to each well. Incubate in a 37 °C 5% CO_2_ cell culture incubator for 1 h, shaking the cell culture dish every 15 min. After 1 h of incubation, discard the supernatant. Add 1 mL of sterile PBS to each well and wash the plate 2~3 times. Add 2 mL of viral medium per well and place the cell culture dish in a 37 °C cell culture incubator with 5% CO_2_. At various time points after viral infection (h.p.i. = 0, 12, 24, and 48), aspirate the cell supernatants. Add 1 mL of TRIzol to each well, pipette repeatedly to completely exfoliate and lyse the cells, transfer the cell lysate to a 1.5 mL EP tube, and extract the total RNA from the cells according to the Triol instructions.

### 2.4. MiRNA Quantification

To isolate total RNA, both infected and uninfected cells were sampled at different time points and processed with Trizol Reagent (Invitrogen, Thermo Fisher Scientific, USA). To eliminate any potential DNA contamination, the extracted total RNA was treated with RNase-free DNase I (Roche, Sigma, St. Louis, MO, USA). Following this, according to the manufacturer’s protocol, the entire RNA sample was incubated with poly(A) polymerase (PAP) in a 20 μL reaction mixture at 37 °C for 1 h. Using a poly(T) adapter, 1 μg of the total RNA was then reverse-transcribed into cDNA. For the quantification of each miRNA, 50 ng of the synthesized cDNA was used in a 25 μL reaction system. The program was executed as outlined below: the thermal cycling protocol consisted of an initial denaturation at 95 °C for 10 min, followed by 40 amplification cycles comprising three steps: denaturation (95 °C, 30 s), annealing (55 °C, 30 s), and extension (72 °C, 30 s). The reactions were conducted on the ABI7500 Fast instrument (Applied Biosystems, Thermo Fisher Scientific, USA).

### 2.5. MiRNAs and miRNA Transfection

micrON^®^ miRNA mimics of miR-18a-5p and the control were acquired from RiboBio (RiboBio, RiboBio, Guangzhou, China). MiRNA transfection was conducted utilizing the Lipofectamine2000 transfection reagent (ThermoFisher Scientific, USA) in accordance with the manufacturer’s guidelines.

### 2.6. Virus Infection

A549 cells were inoculated with QH1/H5N1 virus at predetermined titers using serum-free DMEM, followed by incubation at 37 °C for 60 min. After viral adsorption, the cells were washed three times with phosphate-buffered saline (PBS) to remove unbound viral particles. The infected cells were then maintained in 0.2 mL of specialized influenza virus growth medium, which consisted of DMEM supplemented with essential components: 1% penicillin–streptomycin antibiotic mixture, 0.2% bovine serum albumin (AMRESCO LLC, Solon, OH, USA), 25 mM HEPES buffer (Life Technologies Corp., Carlsbad, CA, USA), and 1 μg/mL TPCK-treated trypsin (Sigma-Aldrich Co., Ltd., LLC, St. Louis, MO, USA). The culture was incubated at 37 °C with 5% CO_2_ to support optimal viral replication and cell growth.

### 2.7. Infection in Animals

Six-week-old BALB/c mice with SPF and four-week-old chickens with SPF were selected as experimental animals. The mice and chickens were lightly anesthetized with dry ice and infected with 10^4^ PFU of QH1/H5N1 by nasal drops; the mouse infection volume was 50 μL, the chicken infection volume was 200 μL, and the control group was injected in the same way and with the same volume of PBS. Body weight and clinical manifestations were recorded daily.

### 2.8. Pathological Examination

Lung tissues of mice and chickens were harvested and preserved in 4% paraformaldehyde. Lung tissues were fixed in paraffin and sectioned into 5 μm slices. The sections were stained using hematoxylin and eosin (H&E).

### 2.9. Immunofluorescence Tissue Staining

The lung specimens were fixed in 4% paraformaldehyde for subsequent immunofluorescence analysis. Following paraffin embedding, 5 μm tissue sections were prepared and processed through sequential ethanol treatments for deparaffinization. Antigen retrieval was performed using specialized buffer, followed by permeabilization with 0.5% Triton X-100. Viral antigens were detected using a monoclonal antibody targeting the NP protein of the H5N1 influenza virus. Detection was achieved with a secondary goat anti-rabbit antibody conjugated to FITC. Finally, the sections were stained for DNA with DAPI (purchased from Sigma).

### 2.10. Analysis of Cytokines and Chemokines

After infection, the lungs were collected and homogenized on subsequent days. Total RNA samples were extracted using Trizol Reagent. The levels of TNF-a, IFN-γ, IFN-β, IL-1β, IL-6, NP, and MP-1 were measured by SYBR I-based Real-Time Polymerase Chain Reaction. The data were normalized to GAPDH and the values of wild-type mice at 0 days post-infection. The primers employed in this experiment are listed in S1E.

### 2.11. Antibody

NP protein: Influenza A Virus NP antibody, Gen-GTX125989.

NEDD9: NEDD9 Rabbit pAb, A2521.

β-Actin: β-Actin Rabbit mAb (High Dilution), AC026.

Secondary antibody: HRP-conjugated Goat anti-Rabbit IgG (H + L), AS014.

Secondary antibody: HRP-conjugated Goat anti-Mouse IgG (H + L), AS003.

### 2.12. Western Blotting

Protein samples were isolated from various sources using RIPA lysis buffer, followed by separation through 10% SDS-PAGE. Electrophoretically resolved proteins were transferred to a nitrocellulose membrane (Pall, Port Washington, NY, USA), which subsequently underwent TBST washing. The membrane was then blocked with 5% skim milk (*w*/*v*) prior to sequential incubation with specific primary and corresponding secondary antibodies. The enhanced chemiluminescence detection reagent (Genview, Houston, TX, USA) was employed to detect the proteins, and grayscale analysis was conducted using Image J software (Java 1.6.0).

### 2.13. Luciferase Reporter Gene Assay

According to Targetscan prediction, the wild-type sequence spanning positions 338–345 of the 3′UTR of NEDD9 contains the binding site for miR-18a-5p. The mutant sequence, devoid of this binding site, along with the wild-type sequence, was cloned into the pmir-GLO vector. Subsequently, Gene Pharma Shanghai developed the wild-type reporter vector (WT) and the mutant reporter vector (MUT). Subsequently, miR-18a-5p mimics, miR-NC, and pmir-GLO were co-transfected into 293T cells with Lipofectamine™ 3000. After a 48 h incubation period, the cells were lysed on ice for 15 min, followed by centrifugation. Luciferase activity quantification was performed using the Dual Luciferase Reporter Detection System (Promega, Alexandria, NSW, USA) according to the manufacturer’s protocol, with firefly luciferase measurements normalized against renilla luciferase activity.

NEDD9-WT-F:

5′-GGATCTTCCAGATGAGCTCTGTCATCACTGTGATTCACTTATGCT-3′.

NEDD9-WT-R:

5′-CTGCCGTTCGACGATCTCGAGATCTATAGATGATTTGAGGGAACTAGAAGT-3′.

NEDD9-MUT-F:

5′-CTTTCTGGGCGTGGAATAAATTTTCGGAGAGCTACTCATGAT-3′.

NEDD9-MUT-R:

5′-TATTCCACGCCCAGAAAGCTGGCACCCTATATG-3′.

### 2.14. Statistical Analysis

Statistical analyses were performed using GraphPad Prism version 8.0 (GraphPad, Dotmatics, Boston, MA, USA), with data expressed as means ± SDs. Intergroup comparisons were analyzed using the student’s *t*-test, with statistical significance defined as *p* < 0.05. Specific significance levels were denoted as follows: * *p* < 0.05, ** *p* < 0.01, *** *p* < 0.001, and ns (not significant).

## 3. Results

### 3.1. miR-18a-5p Attenuates AIV Replication In Vitro

**Figure 1:**
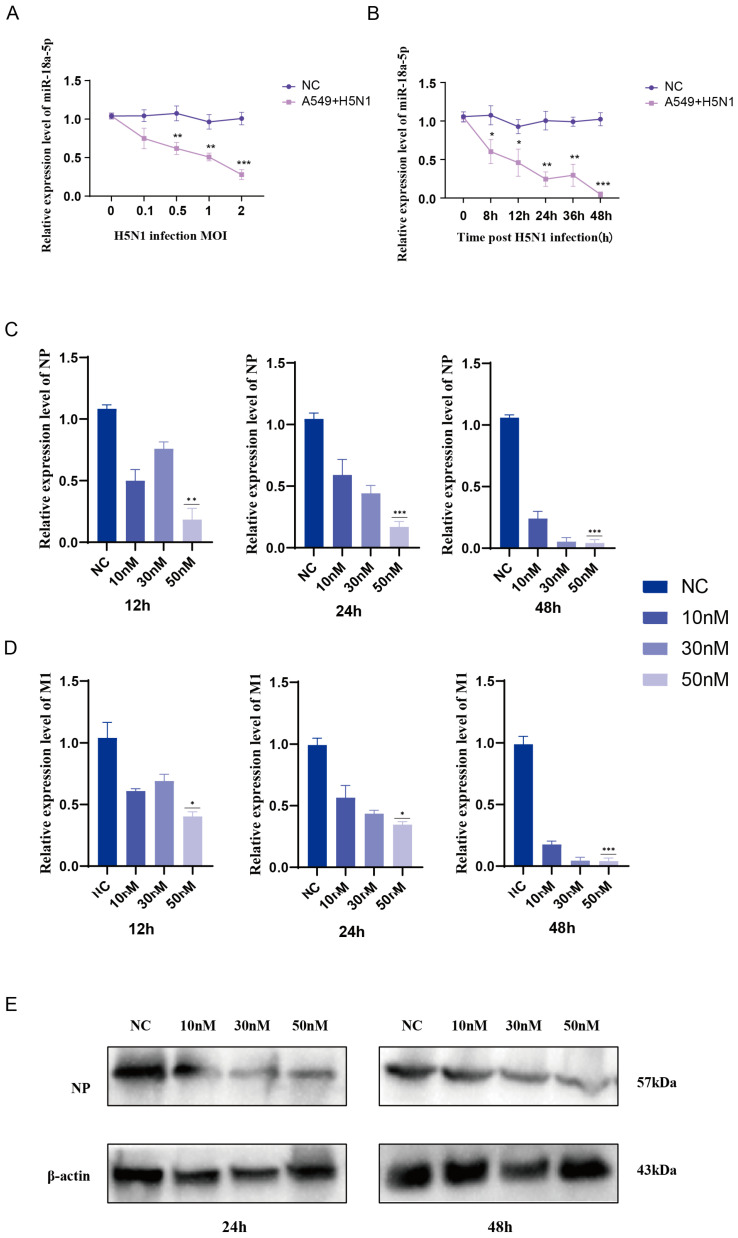
(**A**) Quantitative analysis revealed time-dependent expression patterns of miR-18a-5p following H5N1 virus infection. (**B**) Dose-dependent miR-18a-5p expression was observed in response to H5N1 virus infection, as quantified by qRT-PCR. (**C**) Following transfection with varying concentrations of miR-18a-5p mimic and subsequent QH1/H5N1 infection (24 h.p.i.), NP expression levels were assessed using qRT-PCR. (**D**) M1 expression levels were analyzed by qRT-PCR in A549 cells transfected with miR-18a-5p mimic at different concentrations and infected with QH1/H5N1 at 12, 24, and 48 h.p.i. (**E**) Western blot analysis was performed to evaluate NP expression in miR-18a-5p mimic-transfected A549 cells after QH1/H5N1 infection at 24 and 48 h.p.i. Data are presented as means ± SDs. Asterisks denote the significance levels: * *p* < 0.05; ** *p* < 0.01; *** *p* < 0.001.

MiRNAs play important roles in the interaction between AIV and infected host cells. To explore how HPAI H5N1 influenza virus modulates cellular miRNA expression, we artificially infected a human A549 lung carcinoma cell line with A/environment/Qinghai/1/2008(H5N1) (abbreviated QH1/H5N1) influenza virus. Total RNA samples were isolated from the infected or mock-infected A549 cells, followed by miRNA quantification using real-time Polymerase Chain Reaction (RT-PCR). During the course of infection, H5N1 AIV prominently regulated the expression of cellular miRNAs. At 24 h post infection (h.p.i.), miR-18a-5p was down-regulated with increasing concentrations of QH1/H5N1 (Figure 1A). At MOI = 1 QH1/H5N1 post-infection, miR-18a-5p was down-regulated with increasing duration of infection (Figure 1B). In detail, miR-18a-5p was significantly regulated by QH1/H5N1 in a dose- and time-dependent manner. To explore the influence of miR-18a-5p on QH1/H5N1AIV replication, we transfected A549 cells with mimics of miR-18a-5p prior to infection with QH1/H5N1at MOI = 10, MOI = 30, and MOI = 50. The nucleocapsid protein (NP) and matrix protein (M1) replication of the QH1/H5N1 virus were monitored by measuring RT-PCR at 12 h, 24 h, and 48 h, respectively. As a result, with the extension of infection time, the higher the concentration of the mimic, the more significant the inhibition of influenza protein replication by miR-18a-5p (Figure 1C,D). This was also confirmed by western blotting (Figure 1E), the quantification plot of which is shown in Supplementary Figure S1F.

### 3.2. miR-18a-5p Inhibits the Expression of Pro-Inflammatory Cytokines

**Figure 2:**
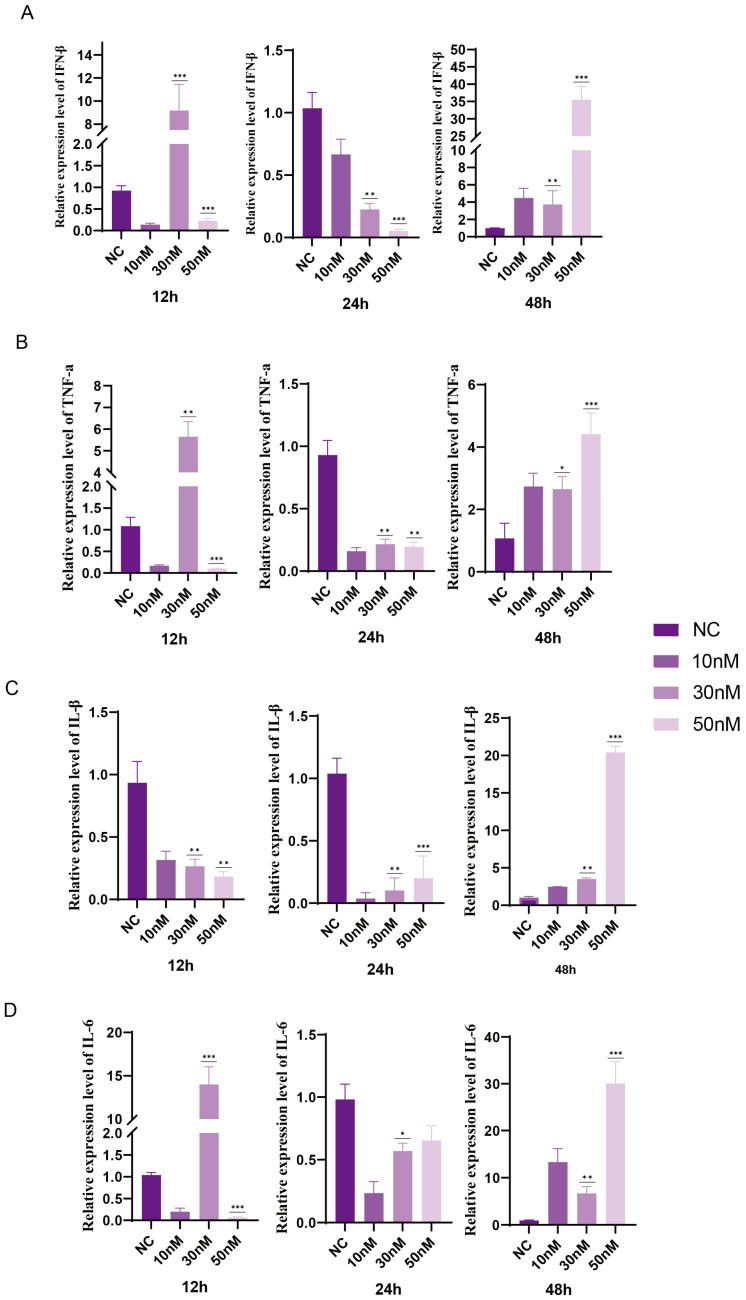
(**A**–**D**). A549 cells were transfected with the different miRNA mimics for 48 h; infected with the QH1/H5N1 virus (MOI = 1) for 12, 24, and 48 h; and subjected to real-time qRT-PCR. (**A**) IFN-β was detected by qRT-PCR. (**B**) TNF-a was detected by qRT-PCR. (**C**) IL-β was detected by qRT-PCR. (**D**) IL-6 was detected by qRT-PCR. Data are presented as means ± SDs. Asterisks denote the significance levels: * *p* < 0.05; ** *p* < 0.01; *** *p* < 0.001.

Through the results at the molecular level and the protein level, we verified that miR-18a-5p inhibits the replication of H5N1 influenza virus; next, we explored the optimal concentration of miR-18a-5p to inhibit the expression of pro-inflammatory cytokines. We transfected A549 cells with 10 nmol, 30 nmol, and 50 nmol of miR-18a-5p mimic, and after 48 h challenged H5N1 with MOI = 1. Cellular RNA was extracted by TRIzol at 12 h, 24 h, and 48 h, and inflammatory cytokines were detected by qRT-PCR. In miR-18a-5p-overexpressing cells, the mRNA expression of IFN-β was significantly increased at 48 h.p.i. (*p* < 0.01) (Figure 2A); the expression of TNF-α was markedly elevated compared to that of the control group (Figure 2B) at 12 (*p* < 0.001), 24 (*p* < 0.01), and 48 h.p.i. (*p* < 0.001) at a concentration of 50 nmol. At the same time, overexpression of miR-18a-5p reduced the expression of IL-6 and IL-β from 12 to 24 h.p.i. (*p* < 0.01) at a concentration of 50 nmol compared to the control group (Figure 2C,D). These results indicated that miR-18a-5p promoted the expression of type I IFN and inhibited the expression of pro-inflammatory factors in H5N1 infection.

### 3.3. miR-18a-5p Attenuates AIV Replication in Mammals

**Figure 3:**
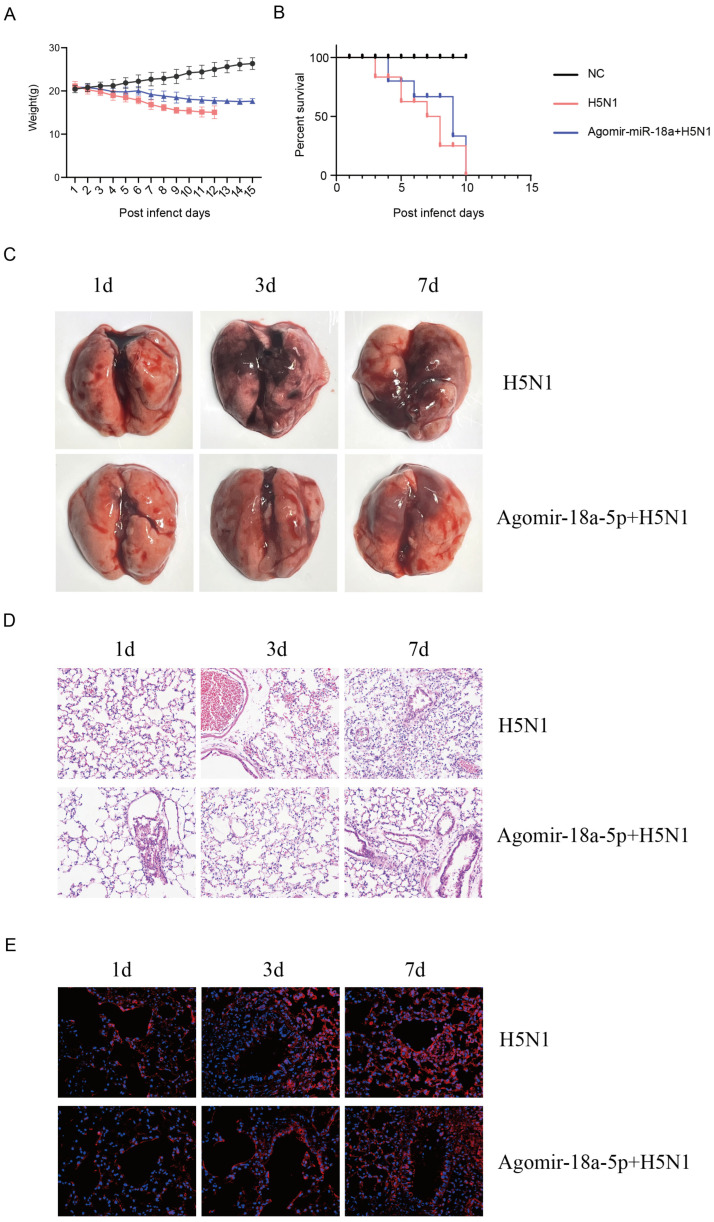
(**A**) Changes in the weight of three babl/c mouse groups (n = 10). (**B**) Survival curves of three babl/c mouse groups (n = 10). (**C**–**E**) From day 1 to day 7 after influenza virus infection in H5N1 mice and agomir-miR-18a-5p + H5N1-treated mice, pathological lesions in the lungs of mice, HE staining, and immunofluorescence.

Having established the inhibitory effect of miR-18a-5p on AIV replication in vitro, we subsequently investigated its potential impact on mortality and morbidity associated with AIV infection in vivo. We used 6-week-old balb/c mice injected with agomir-18a-5p and agomir-NC (n = 10, 80 mg/kg) in the tail vein every three days for a total of three weeks, intranasally inoculated with QH1/H5N1 influenza virus at a dose of 10^4^ plaque forming units (PFU; Supplementary Figure S1A) in the fourth week. The miR-18a-5p in the lungs and PBMCs of the two groups was quantitatively detected (*p* < 0.05) (Supplementary Figure S1B). In contrast, mice in the agomir-NC group exhibited more significant weight loss (Figure 3A,B) and more pronounced clinical manifestations, such as huddling, listlessness, dyspnea, and loss of appetite. As expected, the agomir-NC-infected mice showed more severe and extensive viral pneumonia with substantial damage and inflammation (Figure 3C). The detection of lung NP quantification in mice showed that the lung NP load in the agomir-18a group was lower than that in the NC group (Supplementary Figure S1I). With the increase in infection time, the characteristics of lung tissue necrosis in the NC group were more prominent, showing more severe edema, hemorrhage, inflammatory cell infiltration, and extensive lobar lesions (Figure 3D). We further examined viral NP protein distribution in the lungs. The findings indicated that viral particles were extensively dispersed in the lung tissues of agomir-NC mice. In contrast, in agomir-18a-5p mice, a restricted distribution pattern was observed (Figure 3E).

### 3.4. miR-18a-5p Attenuates AIV Replication in Poultry Animals

**Figure 4:**
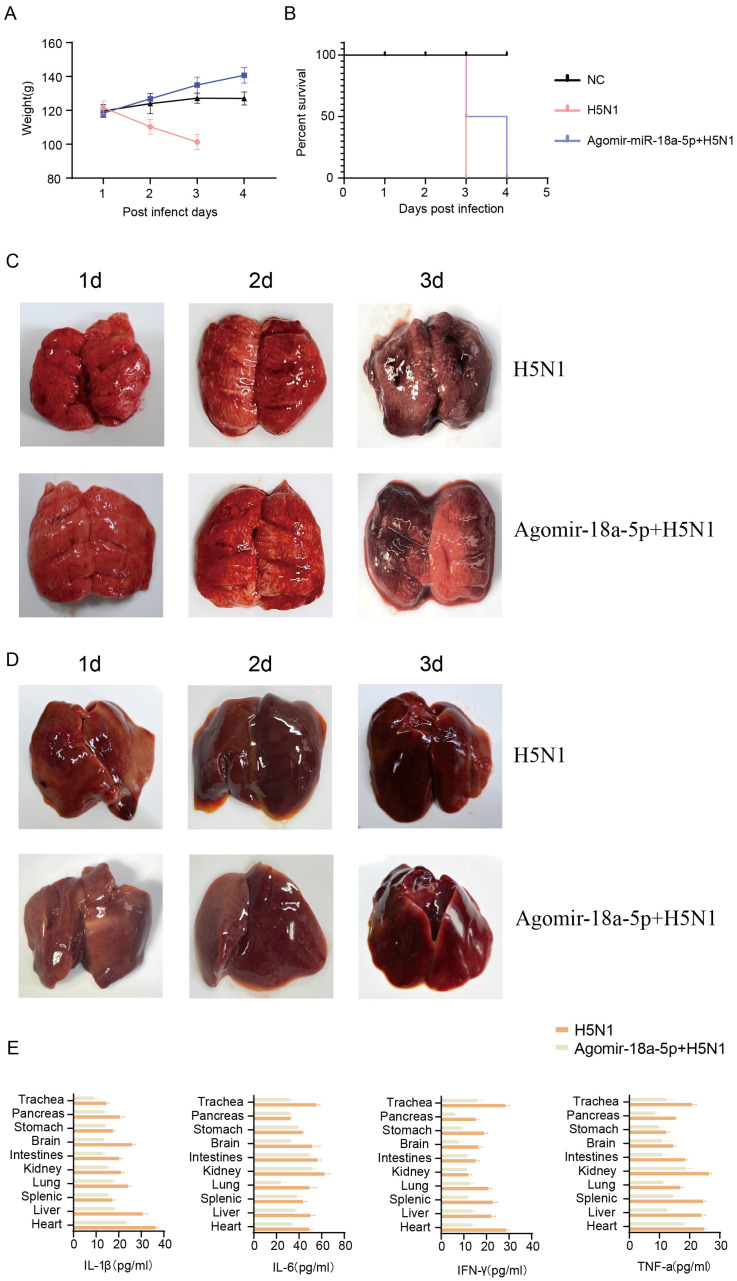
(**A**) Changes in the weight of three SPF chicken groups (n = 3). (**B**) Survival curves of three SPF chicken groups (n = 3). (**C**,**D**) From day 1 to day 3 after influenza virus infection in H5N1 chicken, pathological lesions in the lungs and lives of chicken. (**E**) The expression of inflammatory cytokines in different organs was detected by ELISA in the H5N1 and agomir-miR-18a-5p + H5N1 groups.

Following the demonstration of miR-18a-5p’s inhibitory effect on AIV replication in mammalian systems, we subsequently extended our investigation to explore its potential impact on AIV-induced mortality and morbidity in avian species. We used 4-week-old SPF chicken injected with agomir-18a-5p and agomir-NC, (n = 3, 80 mg/kg) via intravenous injection on the medial side of the wing every three days for a total of three weeks, intranasally inoculated with QH1/H5N1 influenza virus at a dose of 10^4^ plaque forming units (PFU; Supplementary Figure S1A) in the fourth week. The miR-18a-5p in the lungs and PBMCs of the two groups was quantitatively detected (*p* < 0.05) (Supplementary Figure S1C). The results were consistent for infected mice; individuals in the agomir-NC group showed higher levels of weight loss and more severe clinical symptoms (Figure 4A). With the exception of the NC group, the H5N1 group all died on the third day, while the agomir group all died on the fourth day (Figure 4B). We performed autopsies on d.p.i. 1, 2, and 3. As expected, chicks in the H5N1-infected group exhibited more severe and more extensive viral pneumonia with substantial damage and inflammation (Figure 4C); the quantitative detection of NP in chicken lungs showed that the NP load in the agomir-18a group was lower than that in the NC group, which was consistent with the results for mice (Supplementary Figure S1J), with more pronounced liver outcomes (Figure 4D). We further examined the expression of inflammatory cytokines in various organs of chickens; the results clearly showed that the expression of inflammatory cytokines in the H5N1 group was higher than in the agomir-18a-5p group in the organs tested (Figure 4E). The study demonstrated that miR-18a-5p significantly reduces mortality and morbidity in poultry animals infected with the H5N1 influenza virus. Chickens treated with agomir-18a-5p exhibited less weight loss, milder clinical symptoms, and lower mortality compared to the agomir-NC control group. Additionally, miR-18a-5p treatment resulted in reduced viral pneumonia severity, less liver damage, and lower expression of inflammatory cytokines in various organs. These findings suggested that miR-18a-5p effectively enhances the antiviral response, providing a potential therapeutic strategy for combating H5N1 influenza virus infections in poultry.

### 3.5. NEDD9 Is the Target of miR-18a-5p

**Figure 5:**
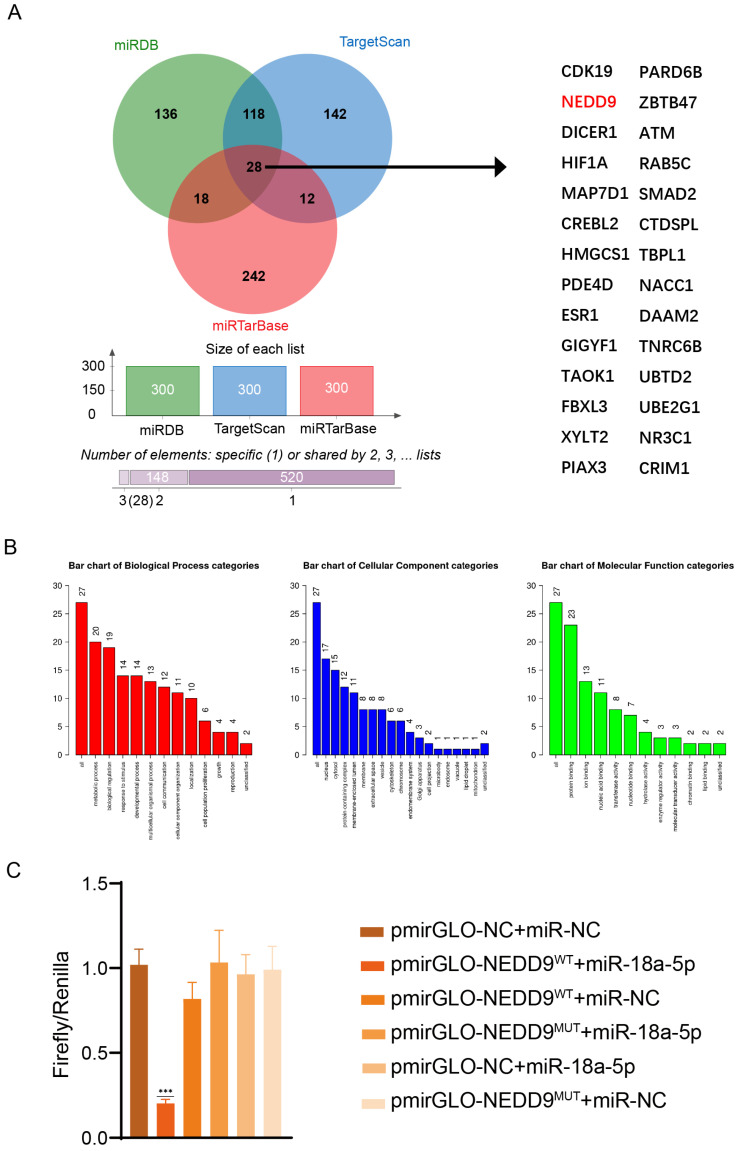
(**A**) Three related websites were used to identify target genes of miR-18a-5p; the first 300 target genes intersected to give 28. (**B**) The online website Web-Gestalt predicted the KEGG pathway for miR-18a-5p. (**C**) Dual luciferase reporter assay for miR-18-5p targeting NEDD9. Data are presented as means ± SDs. Asterisks denote the significance levels:*** *p* < 0.001.

We next tried to find the targets of miR-18a-5p. MiRDB (https://mirdb.org/mirdb/index.html, accessed on 27 February 2025), miRTarBase (https://mirtarbase.cuhk, accessed on 27 February 2025), and TargetScan (https://www.targetscan.org, accessed on 27 February 2025) were used to predict potential mRNAs that could be targeted by miR-18a-5p. The intersections of the top 300 genes in the three databases were taken, and we obtained 28 target genes (Figure 5A). We finally found that NEDD9 was one of the common predicted target genes. NEDD9 is currently studied for its association with the progression of cancer, and studies have shown that NEDD9 is elevated in the plasma of patients after SARS-CoV-2 infection, so we speculated whether NEDD9 is related to the replication process of influenza viruses. GO and pathway enrichment analyses were performed on the 28 intersecting target genes. The WebGestalt (Texas, United States) online tool was employed to analyze and generate a series of biological processes related to these target genes. The biological processes cover diverse categories, such as the regulation of cell growth, cell proliferation, and metabolic processes (Figure 5B). To demonstrate that miR-18a-5p targets the 3′-UTR of NEDD9 mRNA, a luciferase reporter was constructed. We amplified part of the NEDD9 mRNA-3′-UTR containing the seed region of miR-18a-5p by PCR and cloned it into a pmirGLO Dual-Luciferase miRNA Target Expression Vector according to the instructions. As a contrast, the seed region of miR-18a-5p in the reporter vector was mutated into a scramble sequence. In this experiment, pmir-NEDD9^WT^, pmir-NEDD9^MUT^, miR-18a-5p mimic, and miR-NC were transfected into 293T cells, as shown in Figure 5C. Then, we detected the luciferase reporter using the MicroInspector program. The data showed that miR-18a-5p suppressed pmir-NEDD9^WT^ firefly luciferase expression, while pmir-NEDD9^MUT^ firefly luciferase expression showed no significant change. Therefore, we concluded that miR-18a-5p immediately binds to 3′-UTR of the NEDD9 mRNA, thus suppressing NEDD9 transcription.

### 3.6. NEDD9 Promotes Influenza Virus Replication

**Figure 6:**
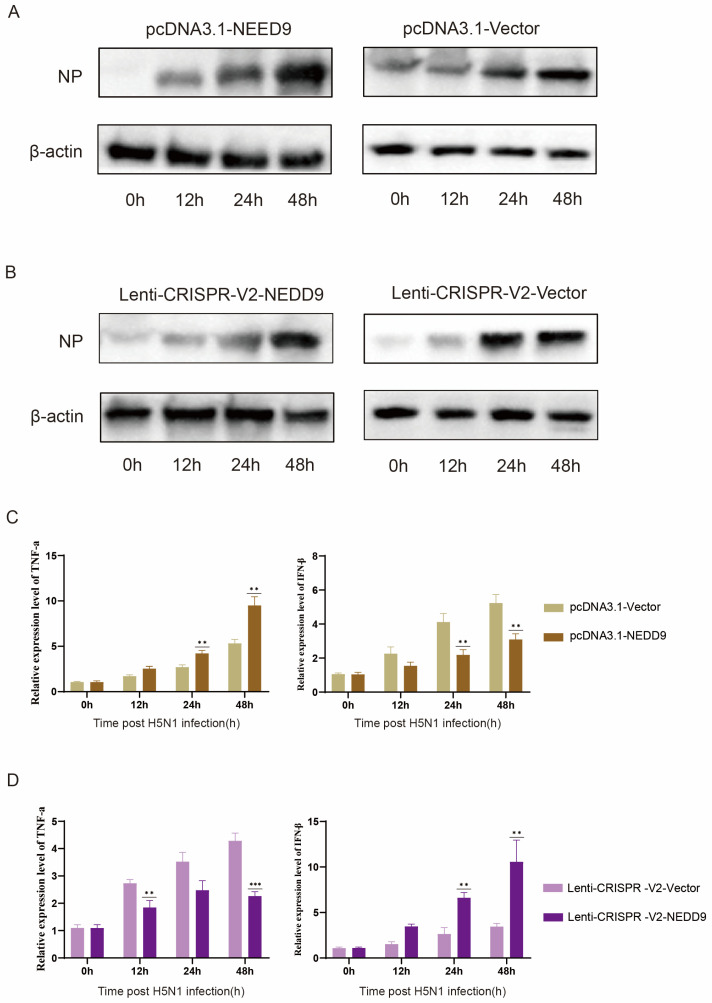
A549 cells were transfected with pcDNA3.1-NEDD9, lenti-crisprv2-NEDD9, or an empty vector for 48 h, followed by QH1/H5N1 infection at 24 h.p.i. (**A**,**B**) NP protein levels were analyzed by Western blotting at various time points post-transfection. (**C**,**D**) qRT-PCR was performed to measure inflammatory factor expression in cells transfected with either pcDNA3.1-NEDD9 or lenti-crisprv2-NEDD9. Data are presented as means ± SDs. Asterisks denote the significance levels:; ** *p* < 0.01; *** *p* < 0.001.

To investigate the potential role of NEDD9 in AIV infection, A549 cells were transfected with pcDNA3.1-NEDD9, lenti-CRISPR-V2-NEDD9, and an empty vector. This led to the successful construction of NEDD9 overexpression and knockout cell lines. Subsequently, these cell lines were infected with QH1/H5N1 for further analysis. Western blot results showed that overexpression of NEDD9 significantly promoted H5N1 replication and peaked at 48 h (Figure 6A) (see the plot in Supplementary Figure S1G), while NEDD9 deletion inhibited the expression of NP (Figure 6B) (see the plot in Supplementary Figure S1H). Inflammatory cytokine expression was evaluated by qRT-PCR at various time points, suggesting that NEDD9 overexpression inhibited the expression of IFN-β (Figure 6C), and NEDD9 knockout significantly inhibited the expression of inflammatory cytokines (Figure 6D). These findings unmistakably defined NEDD9′s function as a viral factor in the cellular reaction to H5N1 infection.

## 4. Discussion

During the course of this research, we revealed the following: (1) The expression of miR-18a-5p was detected to be down-regulated both in vivo and in vitro, and its induction was demonstrated to be directly mediated by H5N1. (2) MiR-18a-5p plays a functional role in protecting the lungs and improving survival in response to AIV. (3) MiR-18a-5p may inhibit the replication of QH1/H5N1 virus by inhibiting the expression of NEDD9. Considering the fact that miRNAs are widely conserved between mammals and birds [28], miR-18a-5p may be particularly well-suited as a possible therapeutic target for avian influenza H5N1. This is due to the fact that miR-18a-5p plays a function in the protection of mouse lungs.

To initiate the study, we utilized public databases, including miRTarBase, miRDB, and TargetScan, to predict the target genes of miR-18a-5p. This allowed us to determine the functionality of the genes that are intended to be targeted by miR-18a-5p. Following the intersection of the first 100 target genes from the aforementioned databases, a total of 28 target genes of miR-18a-5p were found. The WebGestalt website was utilized for the purpose of conducting KEGG pathway and GO functional enrichment analyses. “Cell regulation” and “cell response to stimulus” were found to be connected with a portion of the enriched pathways, as demonstrated by the findings of the KEGG analysis. Additionally, in order to further analyze the miR-18a-5p target that is involved in the regulation of H5N1, we also examined the intersection of the three expected findings using a Venn diagram. We discovered that NEDD9 was one of the projected target genes that was shared by all of the predicted targets. By performing a luciferase experiment, we were able to demonstrate that miR-18a-5p targets *NEDD9* in a direct manner. Through the use of Western blot tests, we were able to demonstrate that miR-18a-5p has the ability to directly reduce the amount of *NEDD9* protein in the body.

MiR-18a belongs to the miR-17∼92 miRNA cluster (encoding miR-17, -18a, -19a, -20a, -19b-1, and -92a-1) [29]. Haroun et al. [30] showed that reduced expression levels of hsa-mir-18a-5p may be a poor prognostic marker for COVID-19; however, the pathogenic role of miR-18a-5p in the virus remains unknown. miR-18a-5p mimics inhibited the replication of influenza protein NPs (Figure 1C,E) and the expression of inflammatory cytokines (Figure 2) in A549 cells. It was further confirmed in mice and chickens that miR-18a-5p mimics reduce mortality and inhibit weight loss, as well as reducing lung damage (Figure 3 and Figure 4). Our results suggested that the prolongation of miR-18a-5p mimics during adaptation does provide sustained benefits. On days 1 and 7, we observed that the miR-18a-5p mimetic significantly reduced lung damage and reduced mortality, confirming the inhibition of influenza virus by miR-18a-5p.

Recent investigations have revealed a significant association between SARS-CoV-2 post-acute sequelae (PASC) and increased circulating NEDD9 levels, with this prothrombotic marker demonstrating an inverse relationship with pulmonary vascular function parameters [31,32]. Therefore, we hypothesize that the role of NEDD9 in regulating cellular processes such as cytoskeletal dynamics, cell adhesion, and immune signaling may indirectly affect viral entry, replication, and immune evasion [33], and we found that the expression of NEDD9 protein was increased in the infection group compared with A549 without H5N1 infection, which was similar to the increase in NEDD9 expression in patients with SARS-CoV-2 infection [34]. The expression of H5N1 influenza protein NP was found to be up-regulated by overexpression of NEDD9 in A549. However, the knockdown of NEDD9 decreased the expression of influenza protein NP (Figure 6). If miR-18a-5p targets NEDD9 or related pathways, it may disrupt the use of viruses such as H5N1 for infection. This indirect link highlights the potential of miR-18a-5p as a therapeutic target for a variety of viruses by modulating host factors critical to the viral life cycle.

## 5. Conclusions

Our results suggest that miR-18a-5p is an important regulator of host cell defense against influenza virus infection. miR-18a-5p inhibits the replication of H5N1 virus by targeting NEDD9 and inhibits the secretion of inflammatory cytokines, and, given the correlation between NEDD9 and respiratory viruses, ongoing studies may help to further elucidate the mechanism of action.

### The Limitations of This Research

Lack of depth in mechanistic research: although miR-18a-5p has been confirmed to inhibit H5N1 viral replication by targeting NEDD9, the specific molecular mechanism of NEDD9 in viral infection has not been fully elucidated. For example, how NEDD9 regulates the expression of the viral protein NP and its interaction with host cell signaling pathways still needs further investigation. Challenges in clinical translation: although miR-18a-5p has shown good protection in animal models, its safety and efficacy in humans have not been validated. miRNA therapeutics still face many challenges in clinical applications, such as delivery efficiency, stability, and potential off-target effects.

In summary, although this study provides an important basis for miR-18a-5p as an antiviral therapeutic target, further research is still needed to overcome the above limitations and promote its translation into clinical applications.

## Data Availability

The authors confirm that the data supporting the findings of this study are available within the article [and/or] its Supplementary Materials.

## Supplementary Materials

The following supporting information can be downloaded at: https://www.mdpi.com/article/10.3390/vetsci12030240/s1.
