# Supplementary material for: Mechanisms of miR-18a-5p Target NEDD9-Mediated Suppression of H5N1 Influenza Virus in Mammalian and Avian Hosts
Source: Vet Sci. 2025 Mar 3;12(3):240. doi: 10.3390/vetsci12030240 (PMC11945371; doi:10.3390/vetsci12030240)
Supplement: Supplementary file 1 [file vetsci-12-00240-s001.zip › Supplementary material 1.pdf]

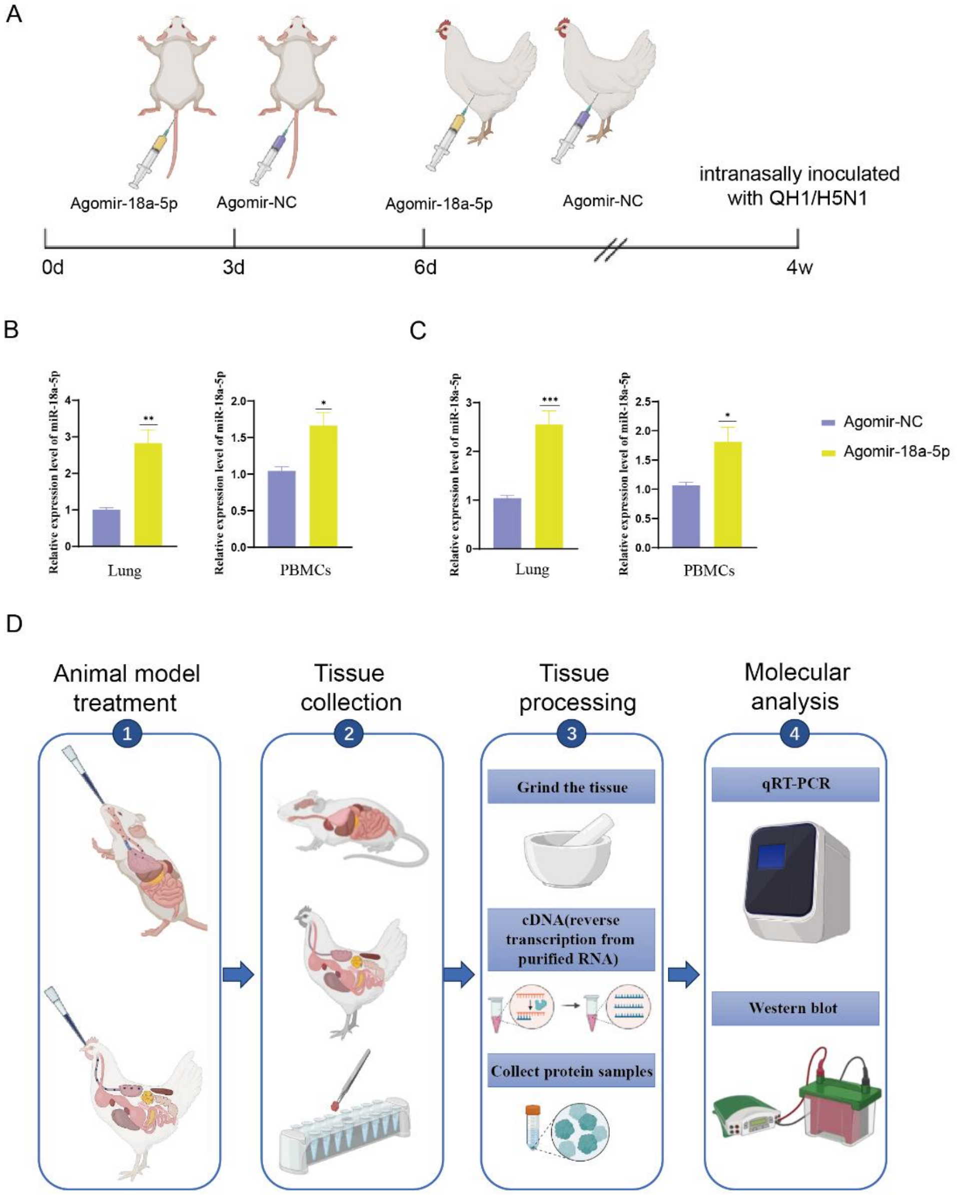

E

Targets	Primer sequences
IFN-β	F: CTCTAGCACTGGCTGGAATGAA<br>R: CCGGAGGTAATCTGTAAGTCTGTT
IL-6	F: TGGCTACTGCCTTCCCTACC<br>R: CAGAGATTTTGCCGAGGATGT
TNF-a	F: CGCATCGCCGTCTCCTACCA<br>R: TGCCCAGATTCAGCAAAGTCCAG
IL-β	F: CAACCAACAAGTGATATTCTCCATG<br>R: GATCCACACTCTCCAGCTGCA
miR-18a-5p	F: 5'-GATAGCAGCACAGAAATATTGGC−3'<br>R: 5'-GTGCAGGGTCCGAGGT−3'
U6	F: 5'-GCTTCGGCAGCACATATACTAAAAT−3'<br>R: 5'-CGCTTCACGAATTTGCGTGTCAT−3'

F

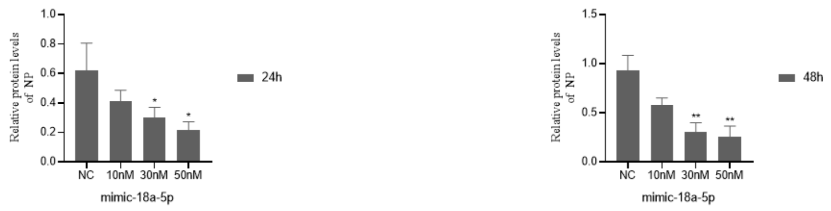

G

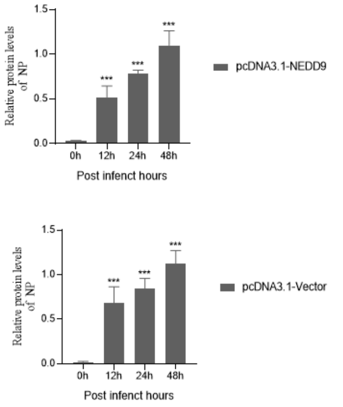

H

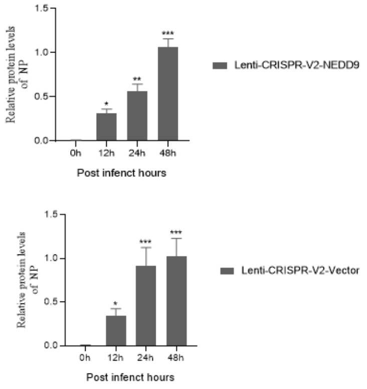

I

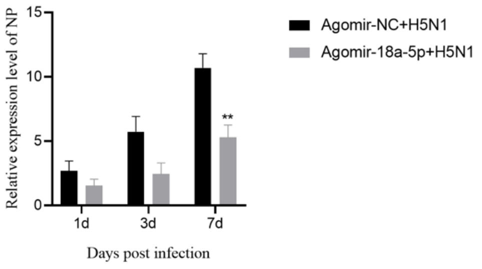

J

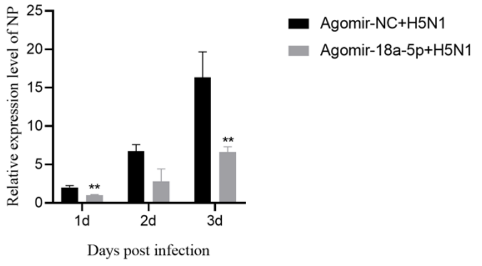

Figure S1. A. Method for injection of miR-18a-5p in model animals. **B**. Expression levels of miR-18a-5p in mouse lungs and PBMCs. **C**. Expression levels of miR-18a-5p in chicken lungs and PBMCs. **D**. Tissue treatment and subsequent test steps after challenge to model animals. **E.** Primer sequence. **F**. Figure 1E western blots quantification plot. **G**. Figure 6A western blots quantification plot. **H**. Figure 6B western blots quantification plot. **I**. Expression of NP in the lungs of QH1/H5N1-infected mice. **J**. Expression of NP in the lungs of QH1/H5N1-infected chickens.
