# Supplementary material for: Mechanisms of miR-18a-5p Target NEDD9-Mediated Suppression of H5N1 Influenza Virus in Mammalian and Avian Hosts
Source: Vet Sci. 2025 Mar 3;12(3):240. doi: 10.3390/vetsci12030240 (PMC11945371; doi:10.3390/vetsci12030240)

Figure 1E

24h-NP

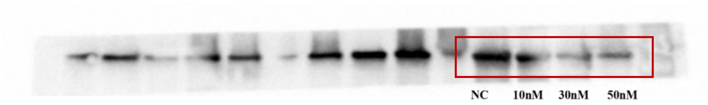

Repeat

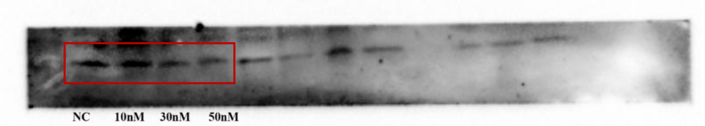

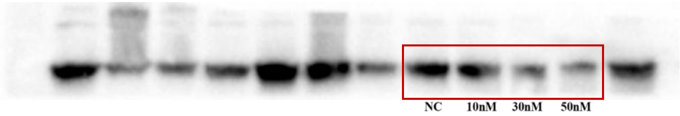

48h-NP

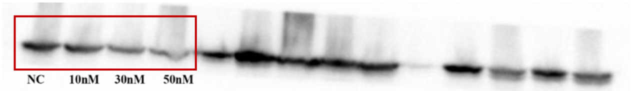

Repeat

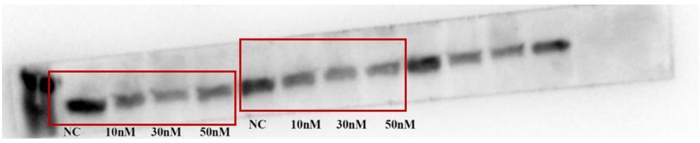

24h-actin

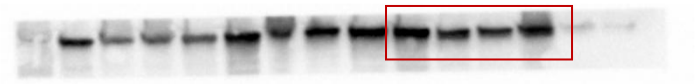

Repeat

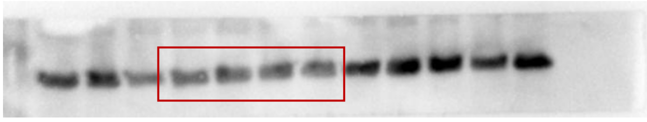

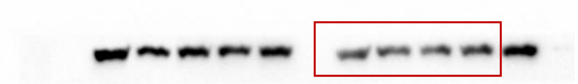

48h-actin

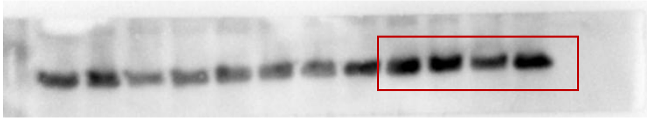

Repeat

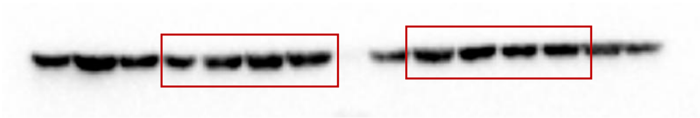

Figure 6a

pcDNA3.1-NEDD9-NP

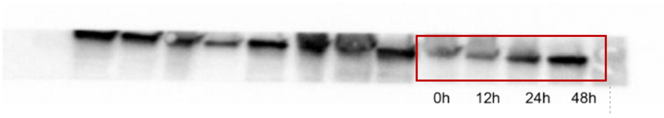

Repeat

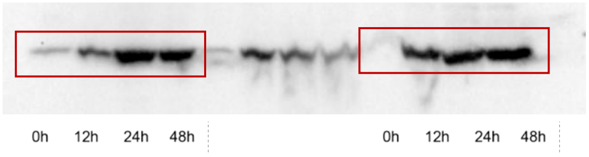

pcDNA3.1-vector-NP

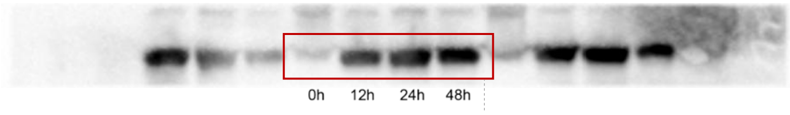

Repeat

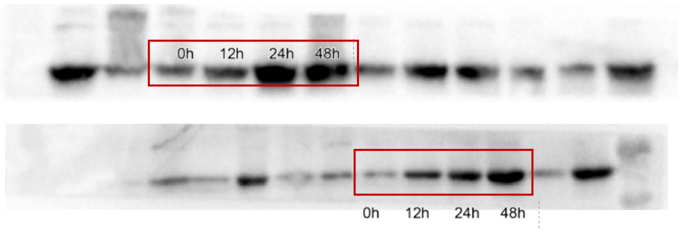

pcDNA3.1-NEDD9-actin

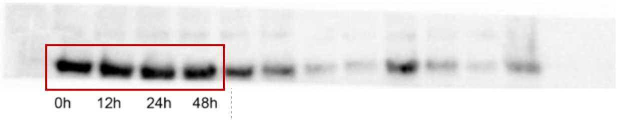

Repeat

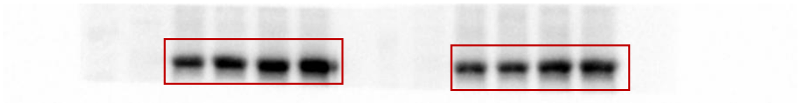

pcDNA3.1-vector-actin

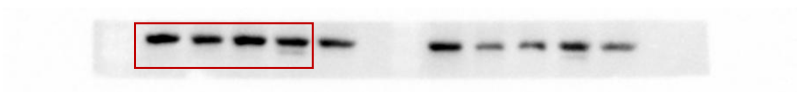

Repeat

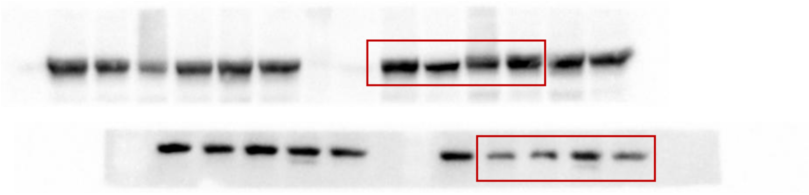

Figure 6B

Lenti-crispr-v2-NEDD9-NP

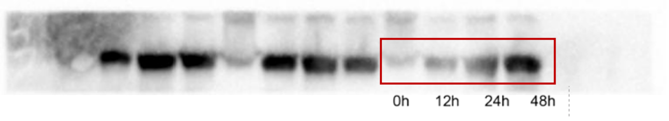

Repeat

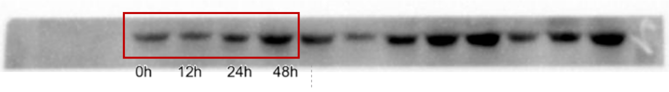

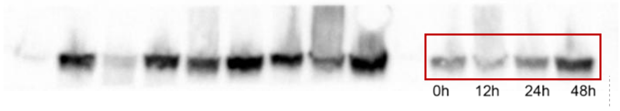

Lenti-crispr-v2-vector-NP

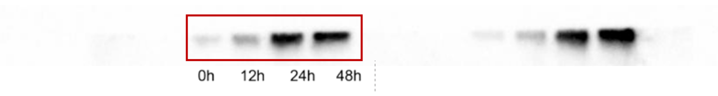

Repeat

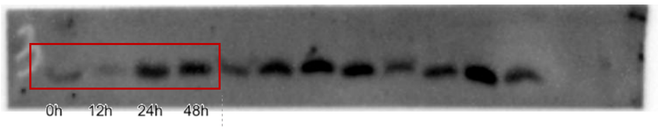

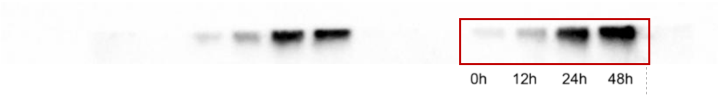

Lenti-crispr-v2- NEDD9-actin

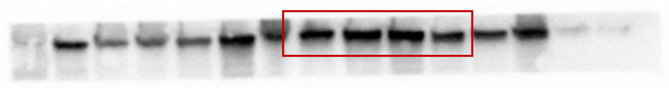

Repeat

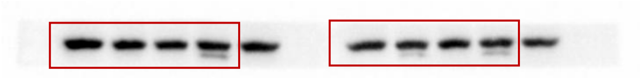

Lenti-crispr-v2-vector-actin

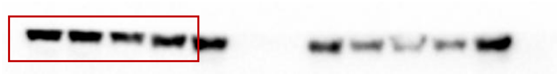

Repeat

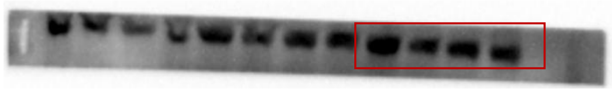

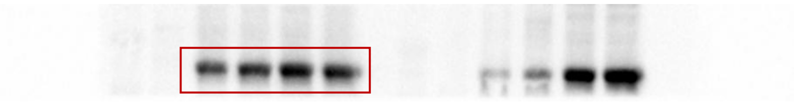

Supplement: Supplementary file 1 [file vetsci-12-00240-s001.zip › Supplementary material-western blot.pdf]
